# Supplementary material for: Fully-exposed Pt-Fe cluster for efficient preferential oxidation of CO towards hydrogen purification
Source: Nat Commun. 2022 Nov 10;13:6798. doi: 10.1038/s41467-022-34674-y (PMC9649773; doi:10.1038/s41467-022-34674-y)
Supplement: Supplementary file 1 — Supplementary Information [file 41467_2022_34674_MOESM1_ESM.pdf]

Supplementary Information

Fully-exposed Pt-Fe cluster for efficient preferential oxidation of CO towards hydrogen purification

*Zhimin Jia[1, 2, #], Xuetao Qin,[3 #] Yunlei Chen,[4, 5 #] Xiangbin Cai,[6 #] Zirui Gao,[3] Mi Peng,[3] Fei Huang,[1] Dequan Xiao,[7] Xiaodong Wen,[4, 5] Ning Wang,[6] Zheng Jiang,[8] Wu Zhou,[9] Hongyang Liu[1, 2, *] and Ding Ma[3, *]*

[1] Shenyang National Laboratory for Materials Science, Institute of Metal Research, Chinese Academy of Sciences, Shenyang 110016, P. R. China.

[2] School of Materials Science and Engineering, University of Science and Technology of China, Shenyang 110016, P. R. China.

[3] Beijing National Laboratory for Molecular Sciences, College of Chemistry and Molecular Engineering, Peking University, Beijing 100871, P. R. China.

[4] University of Chinese Academy of Science, No. 19A Yuanquan Road, Beijing 100049, P. R. China.

[5] State Key Laboratory of Coal Conversion, Institute of Coal Chemistry, Chinese Academy of Sciences, Taiyuan 030001, P. R. China.

[6] Department of Physics and Center for Quantum Materials, Hong Kong University of Science and Technology, Clear Water Bay, Kowloon, Hong Kong SAR, P. R. China.

[7] Center for Integrative Materials Discovery, Department of Chemistry and Chemical Engineering, University of New Haven, 300 Boston Post Road, West Haven, Connecticut 06516, United States

[8] Shanghai Institute of Applied Physics, Chinese Academy of Sciences, Shanghai 201204, P. R. China.

[9] School of Physical Sciences and CAS Center for Excellence in Topological Quantum Computation, University of Chinese Academy of Sciences, Beijing 100049, P. R. China.

[#] These authors contributed equally to this work.

Corresponding Author

*Email: liuhy@imr.ac.cn; dma@pku.edu.cn

This file includes:

Supplementary Discussion

Supplementary 28 Figures

Supplementary 11 Tables

Supplementary References

1. Supplement Figures and Tables

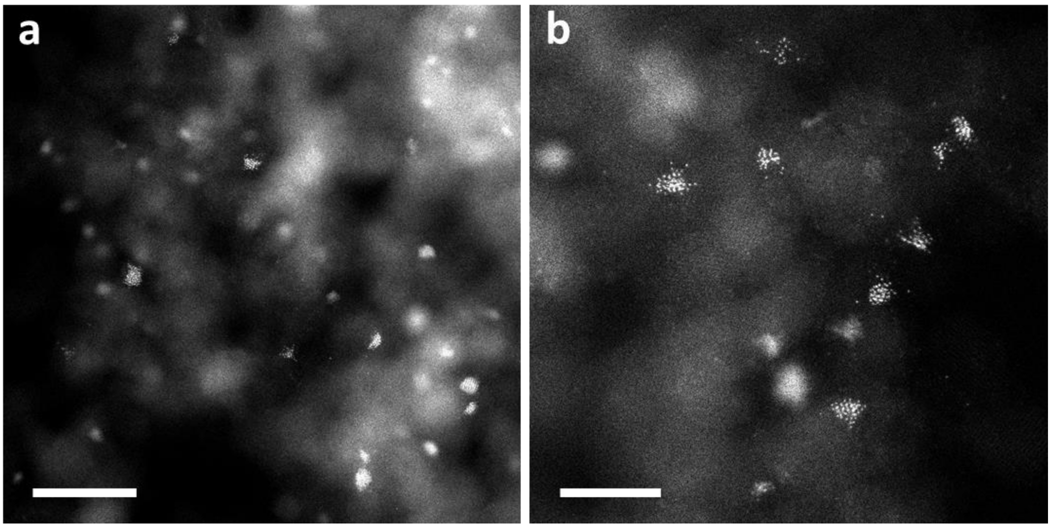

Supplementary Figure 1. Morphology characterization of 0.75Pt0.2Fe/ND@G. HAADF-STEM image of 0.75Pt0.2Fe/ND@G. Scale bar: a, 10 nm; b, 5 nm.

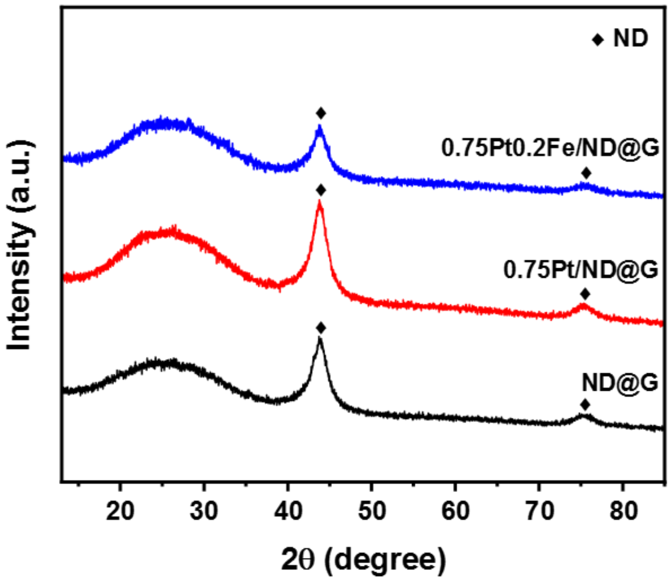

Supplementary Figure 2. XRD characterization of various catalysts. XRD patterns of NDG, 0.75Pt/ND@G and 0.75Pt0.2Fe/ND@G.

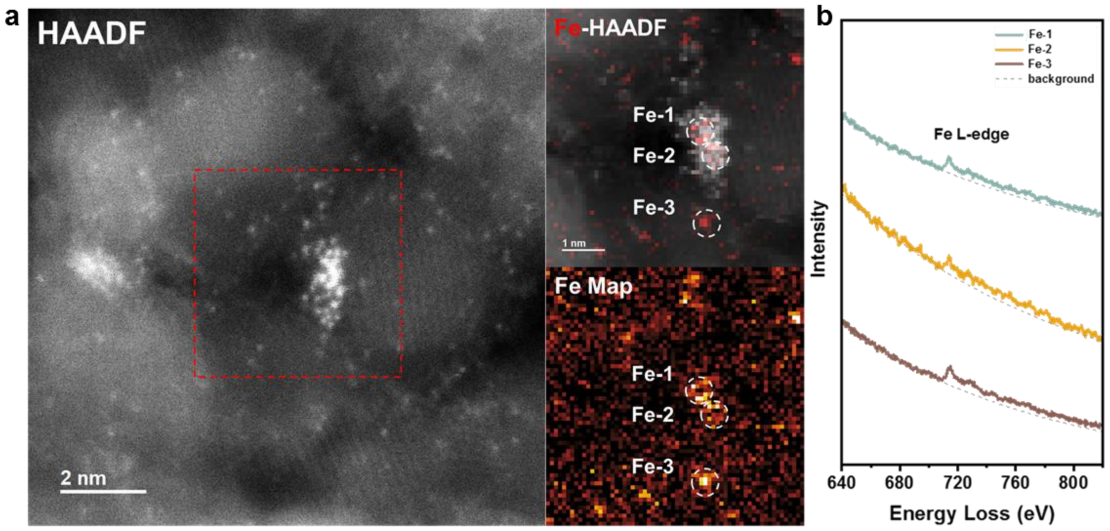

Supplementary Figure 3. EELS characterization of 0.75Pt0.2Fe/ND@G. (a) The HAADF-STEM image of the group of fully-exposed Pt-Fe clusters and the corresponding EELS mapping of Fe element for 0.75Pt0.2Fe/ND@G. (b) EELS spectra of Fe at the corresponding positions marked by white circles in (a).

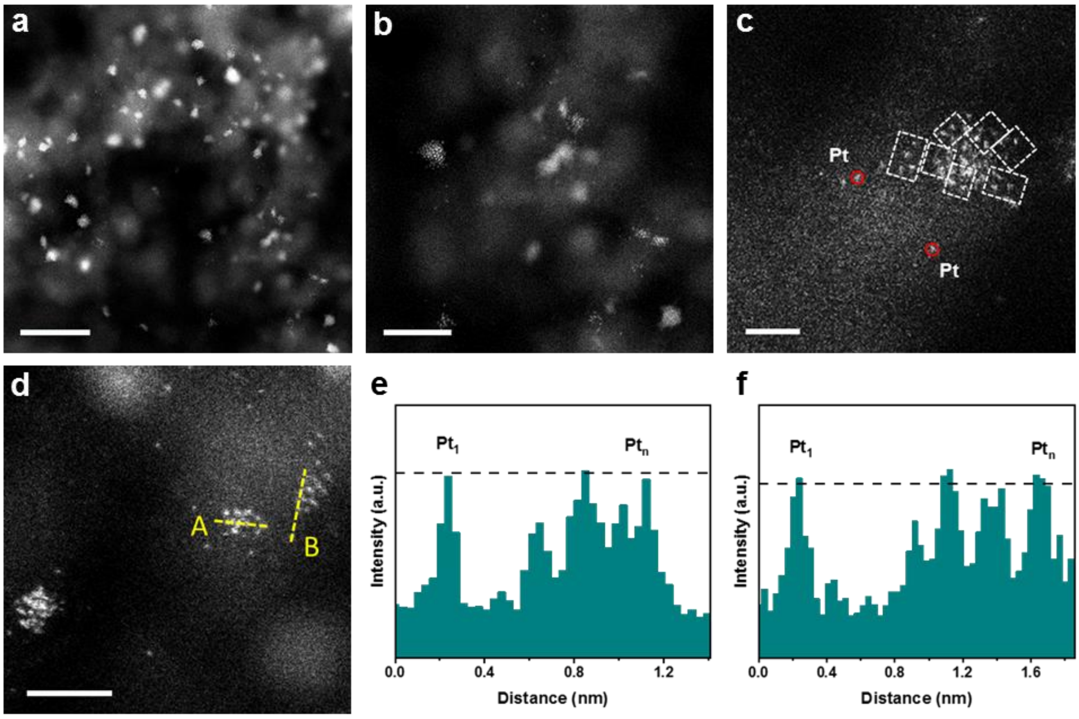

Supplementary Figure 4. Morphology characterization of 0.75Pt/ND@G. HAADF-STEM images of 0.75Pt/ND@G at **(a)** low and **(b-c)** high magnifications. The white and red squares in (c) highlight Pt clusters and atomically dispersed Pt in 0.75Pt/ND@G, respectively. HAADF-STEM image of 0.75Pt/ND@G **(d)** and the intensity profiles along the dashed yellow lines at positions A **(e)** and B **(f)**, respectively. Scale bar: a, 10 nm; b, 5 nm; c, 1 nm; d, 2 nm.

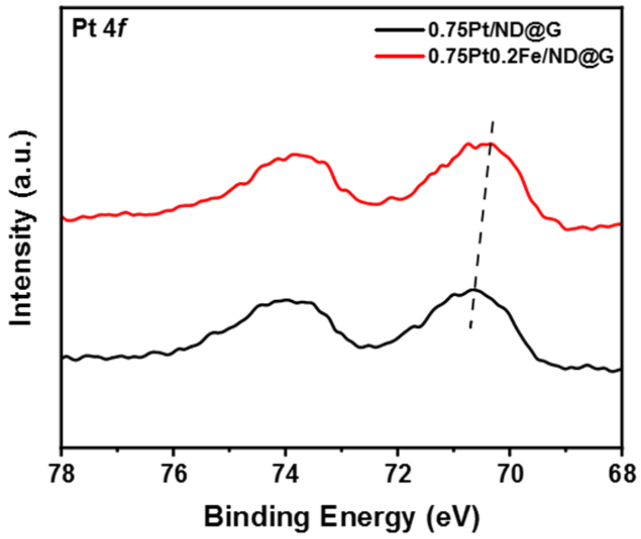

Supplementary Figure 5. XPS characterization of 0.75Pt/ND@G and 0.75Pt0.2Fe/ND@G. XPS spectra of 0.75Pt/ND@G and 0.75Pt0.2Fe/ND@G.

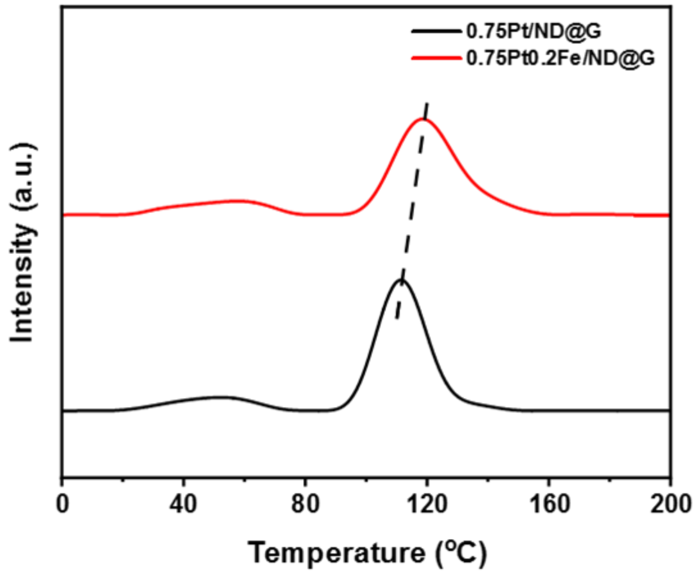

Supplementary Figure 6. Temperature-programmed desorption of CO (CO-TPD) of 0.75Pt/ND@G and 0.75Pt0.2Fe/ND@G. CO-TPD spectra of 0.75Pt/ND@G and 0.75Pt0.2Fe/ND@G.

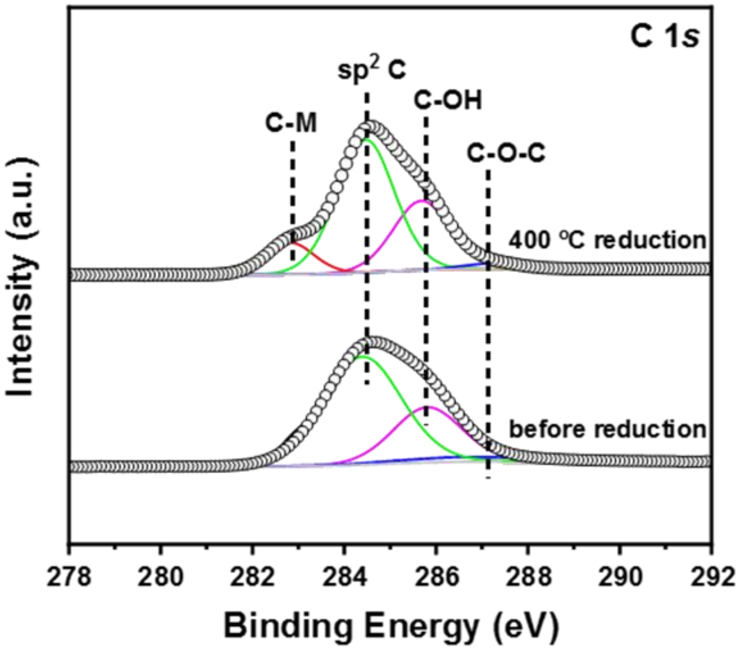

Supplementary Figure 7. XPS characterization of 0.75Pt0.2Fe/ND@G before and after reduction. XPS spectra of 0.75Pt0.2Fe/ND@G catalyst before and after reduction at 400 ºC for C 1*s*.

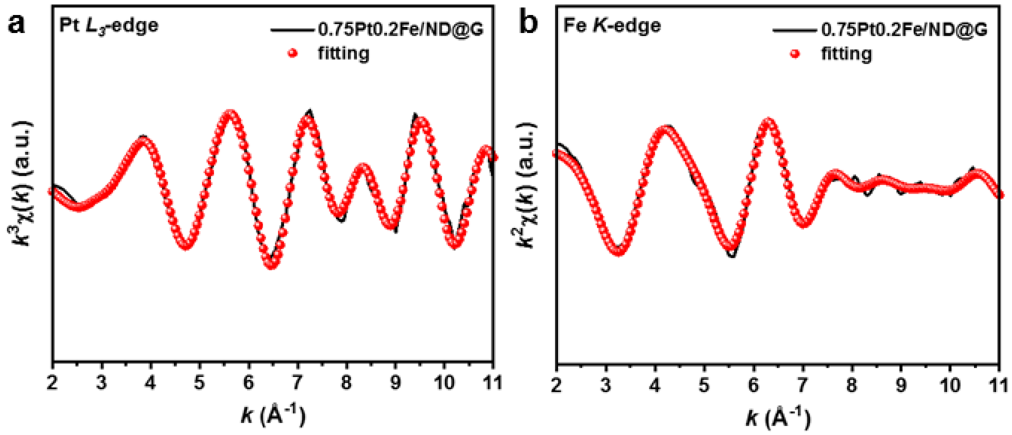

Supplementary Figure 8. The EXAFS fitting at *k*-space of 0.75Pt0.2Fe/ND@G. The corresponding Pt L_3-edge and Fe K-edge EXAFS fitting curves for 0.75Pt0.2Fe/ND@G catalyst at *k*-space (a,b), respectively.

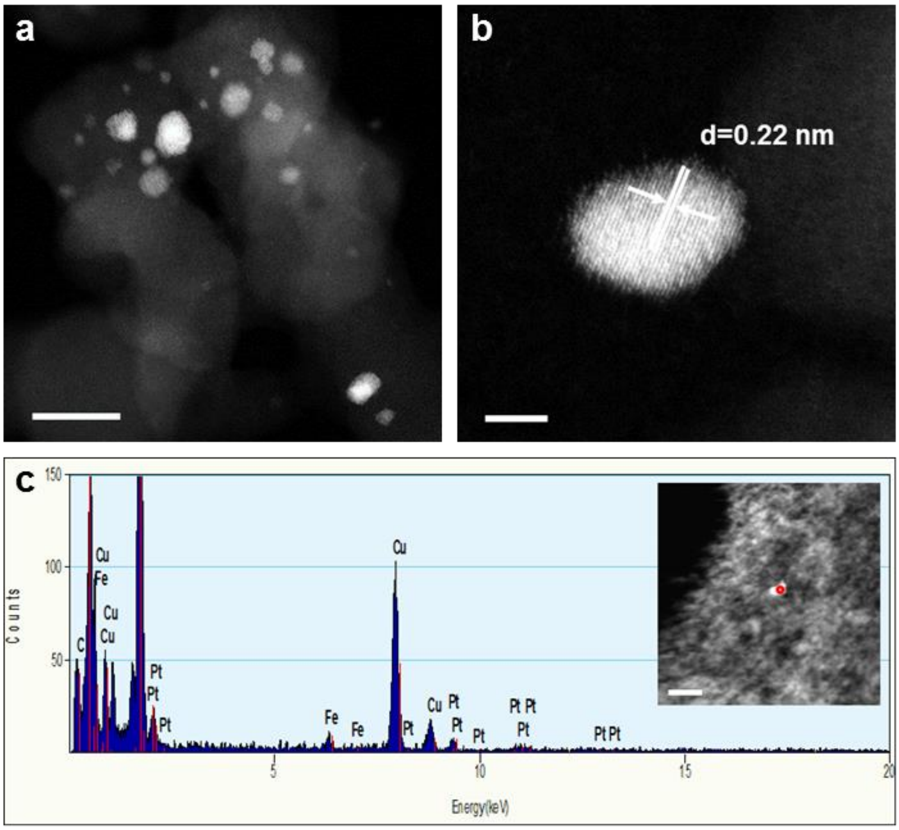

Supplementary Figure 9. Morphology characterization of 0.75Pt0.2Fe/SiO_2. (a,b) HAADF-STEM images of 0.75Pt0.2Fe/SiO_2. **(c)** The corresponding EDS spectroscopy result. Scale bar: a, 10 nm; b, 2 nm; c, 100 nm.

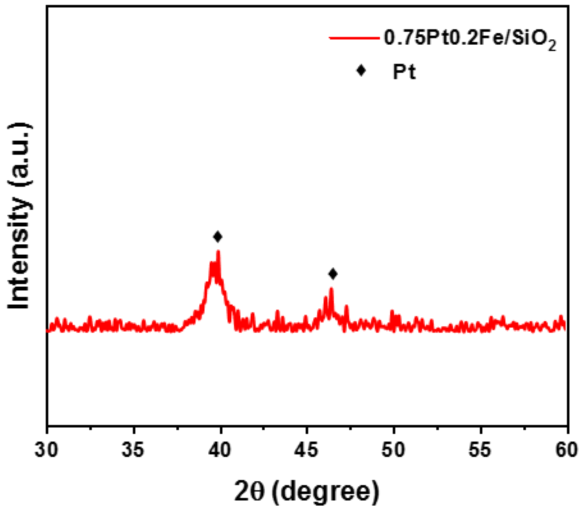

Supplementary Figure 10. XRD characterization of 0.75Pt0.2Fe/SiO_2. XRD pattern of 0.75Pt0.2Fe/SiO_2.

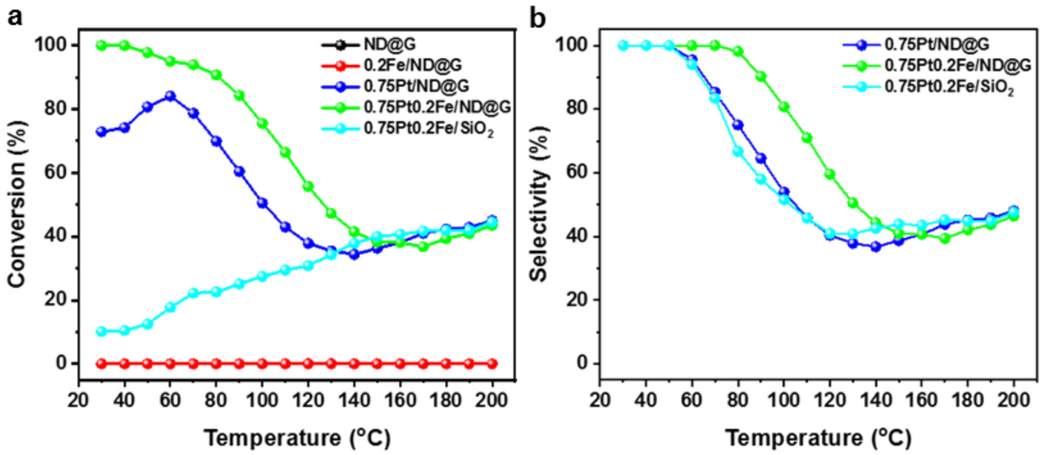

Supplementary Figure 11. Catalytic performance of PROX reaction over various catalysts. CO conversion (a), and selectivity (b) of various catalysts as a function of temperature for PROX reaction. Reaction conditions: 1%CO, 0.5%O_2 and 48% H_2 balanced in He; the space velocity is 45000 mL g^{-1} h^{-1}.

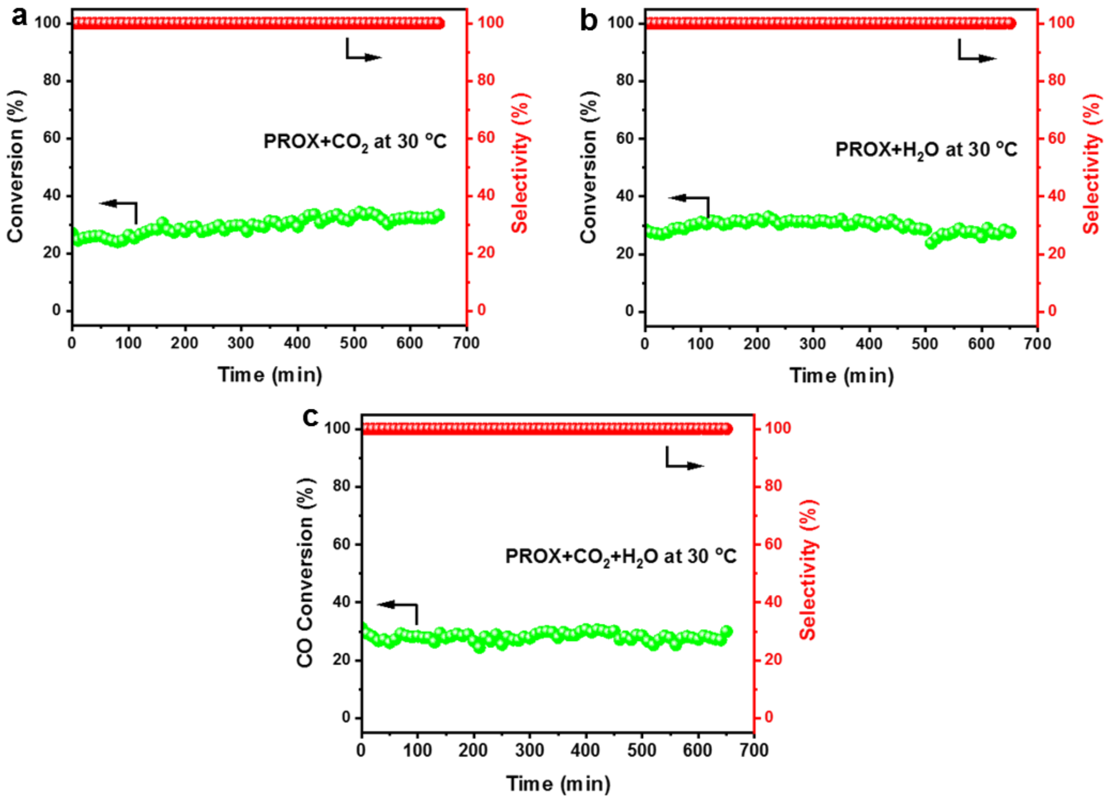

Supplementary Figure 12. The stability of PROX reaction over 0.75Pt0.2Fe/ND@G catalyst. Stability of the 0.75Pt0.2Fe/ND@G catalyst under CO PROX conditions in the presence of CO_2 and H_2O at 30 ºC. Reaction conditions: 1% CO, 0.5% O_2, 48% H_2, and 20% CO_2 **(a)**, 3% H_2O **(b)**, 20% CO_2 and 3% H_2O **(c)**, balanced in helium.

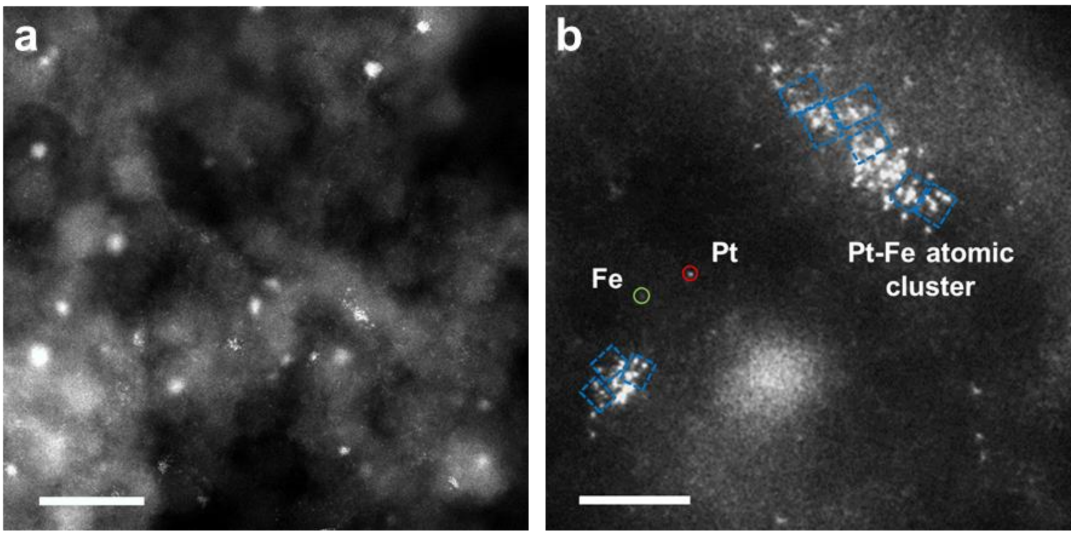

Supplementary Figure 13. Morphology characterization of used 0.75Pt0.2Fe/ND@G. STEM images of the used 0.75Pt0.2Fe/ND@G after PROX reaction. The representative isolated Pt, Fe atoms and fully-exposed Pt-Fe clusters are highlighted by red, green circles and dashed blue rectangles, respectively. Scale bar: a, 10 nm; b, 2 nm.

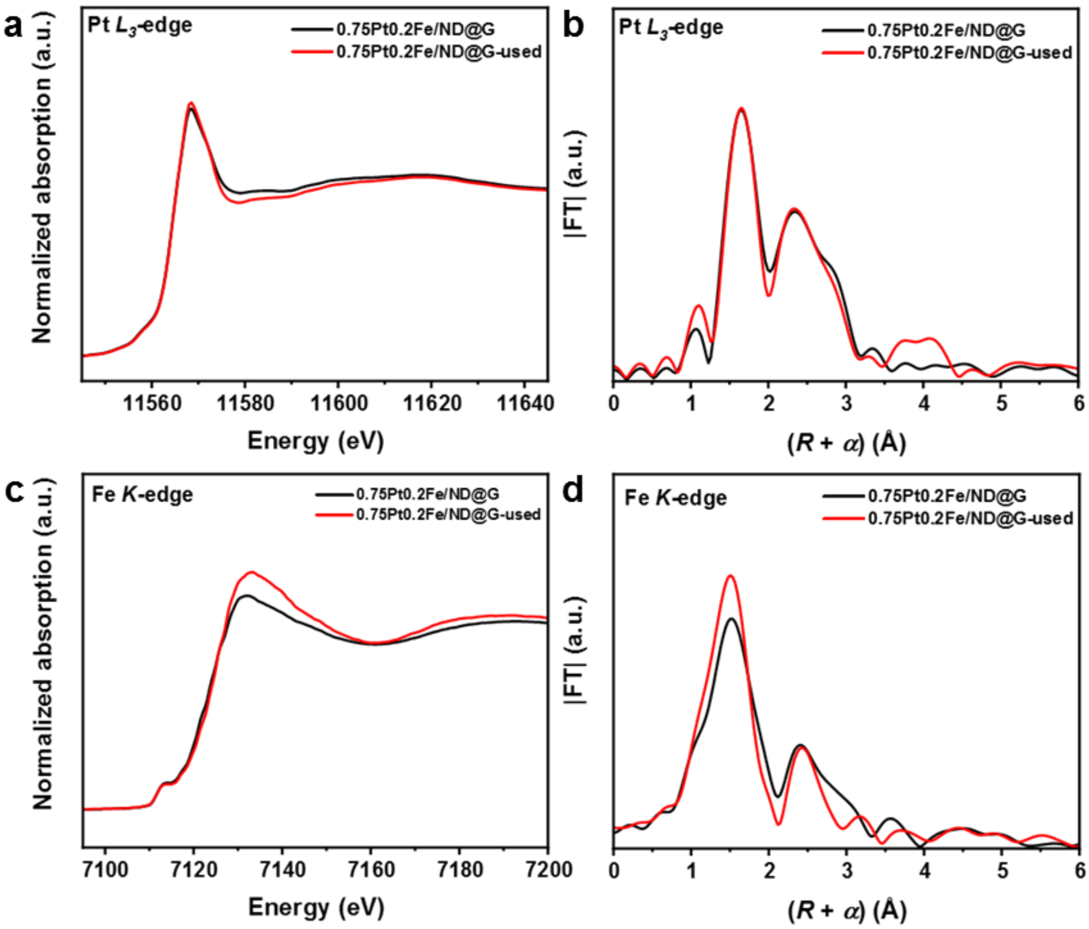

Supplementary Figure 14. X-ray absorption characterization of fresh and used 0.75Pt0.2Fe/ND@G. The normalized X-ray absorption near-edge structure (XANES) spectra **(a)** and Fourier transform extended X-ray absorption fine structure (FT-EXAFS) **(b)** at the Pt L_3-edge for fresh and used 0.75Pt0.2Fe/ND@G; and the normalized XANES spectra **(c)** and FT-EXAFS **(d)** at the Fe K-edge for fresh and used 0.75Pt0.2Fe/ND@G.

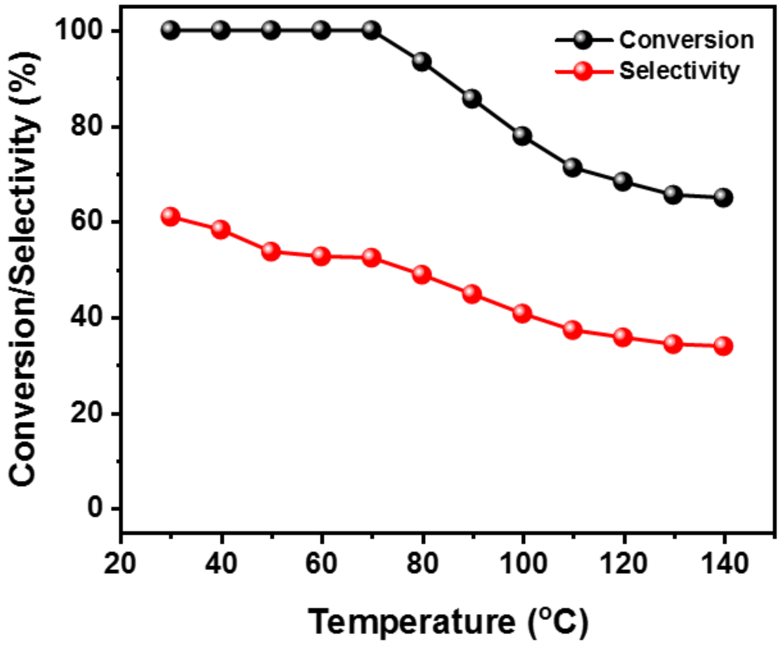

Supplementary Figure 15. Catalytic performance of PROX reaction over0.75Pt0.2Fe/ND@G in the excess oxygen. CO conversion and selectivity of the 0.75Pt0.2Fe/ND@G catalyst in PROX reaction. Reaction conditions: 1%CO, 1%O_2 and 48% H_2 balanced in He; the space velocity is 45000 mL g^{-1} h^{-1}.

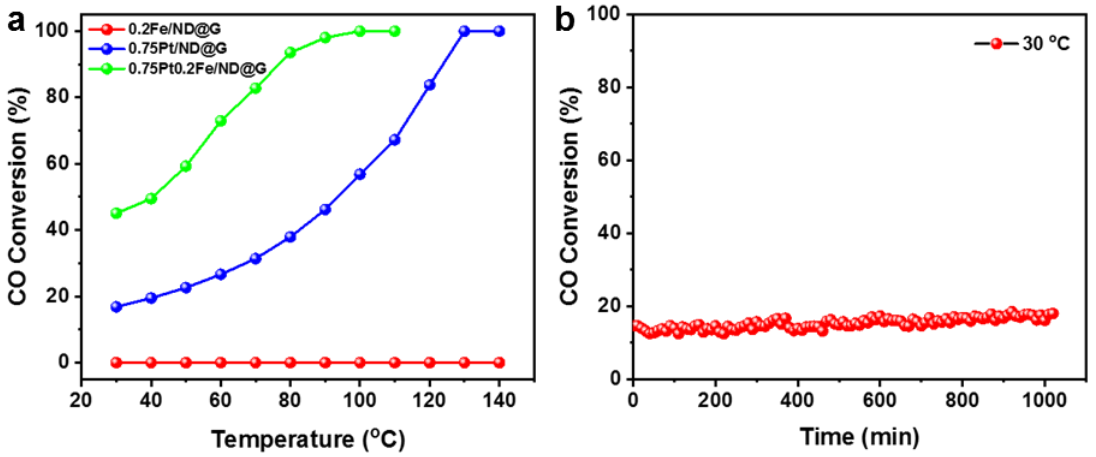

Supplementary Figure 16. Catalytic performance of CO oxidation reaction over various catalysts. (a) CO conversion as a function of reaction temperature over PtFe/ND@G catalysts in CO oxidation reaction (Reaction conditions: 1%CO and 1%O_2 balanced in He; the space velocity is 72000 mL g^{-1} h^{-1}). (b) Stability of the 0.75Pt0.2Fe/ND@G catalyst under CO oxidation conditions (30 ºC, 360000 mL g^{-1} h^{-1}).

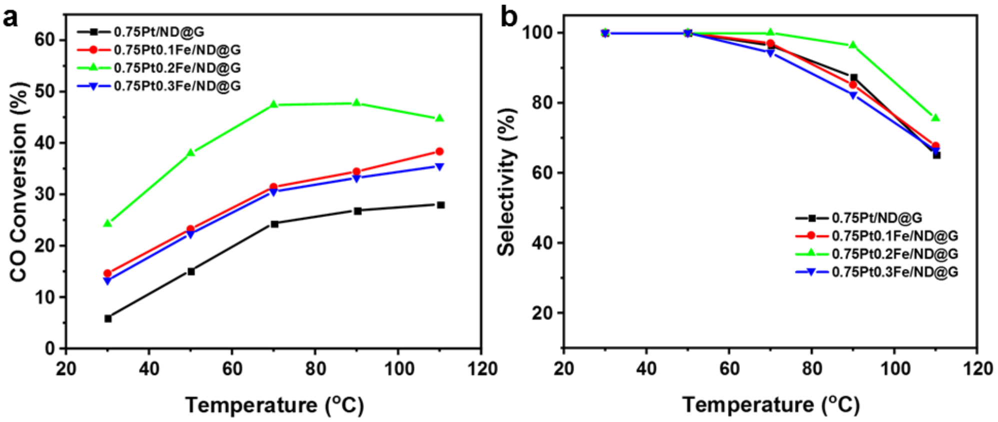

Supplementary Figure 17. Catalytic performance of PROX reaction over various PtFe/ND@G with different Fe loading. CO conversion **(a)** and selectivity **(b)** of various PtFe/ND@G with different Fe loading in PROX reaction. Reaction conditions: 1%CO, 0.5%O_2 and 48% H_2 balanced in He; v=60mL/min.

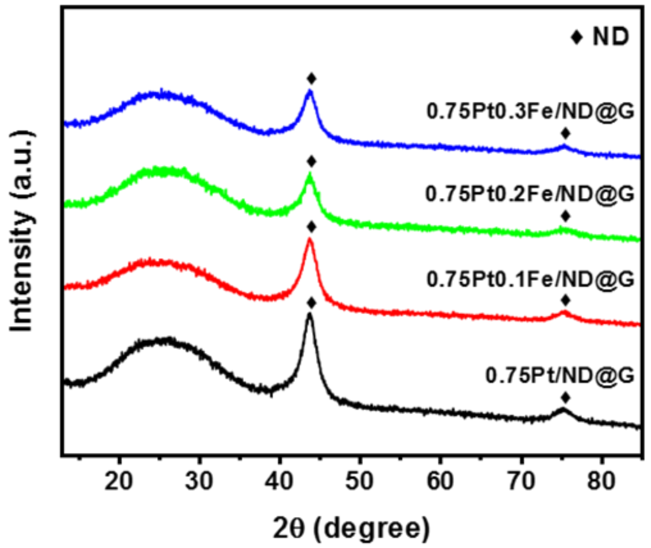

Supplementary Figure 18. XRD characterization of various PtFe/ND@G with different Fe loading. XRD patterns of various PtFe/ND@G with different Fe loading.

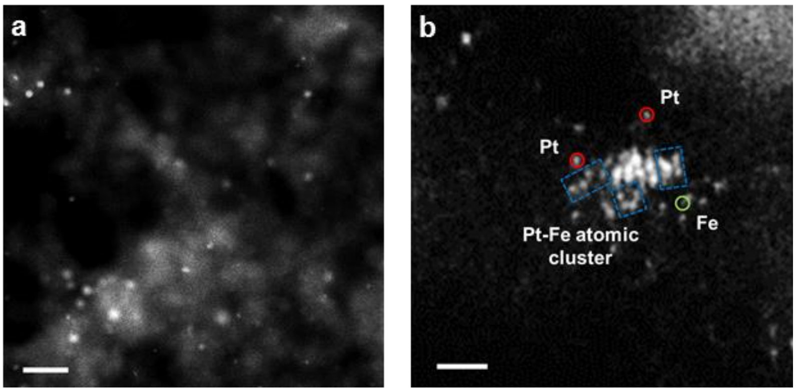

Supplementary Figure 19. Morphology characterization of 0.75Pt0.1Fe/ND@G. The HAADF-STEM images of 0.75Pt0.1Fe/ND@G at low magnification **(a)** and high magnification **(b)** where representative isolated Pt, Fe atoms and fully-exposed Pt-Fe clusters are highlighted by red, green circles and dashed blue rectangles, respectively. Scale bar: a, 10 nm; b, 1 nm.

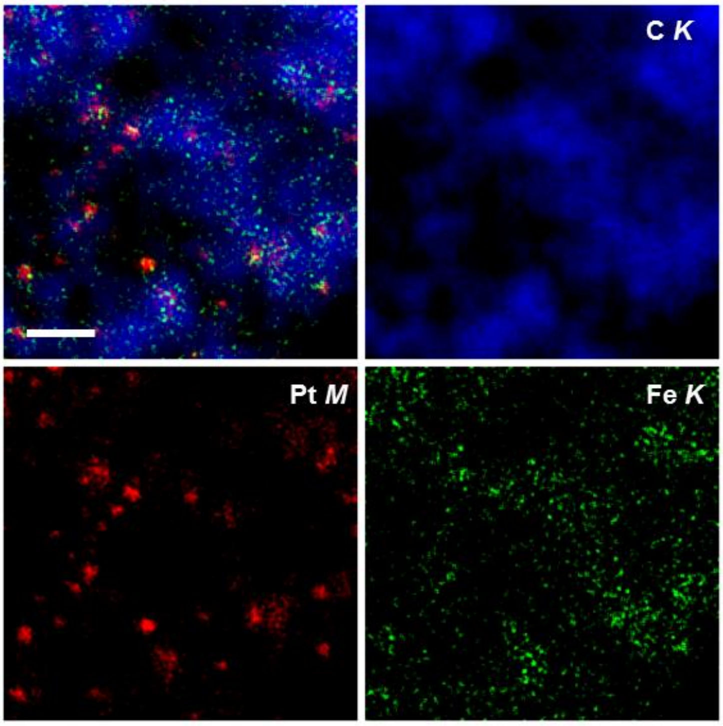

Supplementary Figure 20. EDX characterization of 0.75Pt0.1Fe/ND@G. Energy-dispersive X-ray (EDX) mapping spectroscopy of 0.75Pt0.1Fe/ND@G catalyst, scale bar = 10 nm.

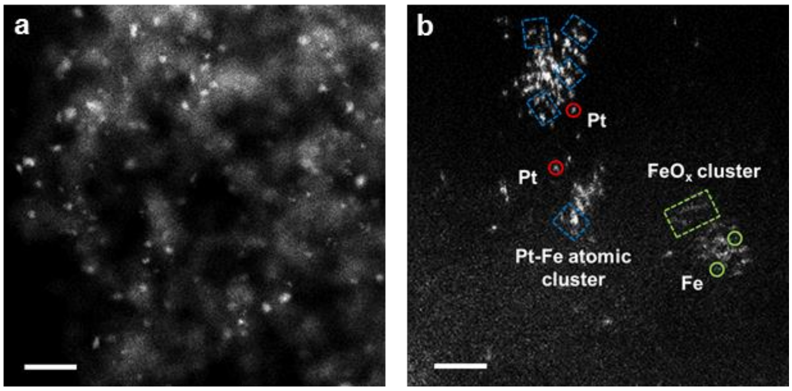

Supplementary Figure 21. Morphology characterization of 0.75Pt0.3Fe/ND@G. The HAADF-STEM images of 0.75Pt0.3Fe/ND@G at low magnification **(a)** and high magnification **(b)** where representative isolated Pt, Fe atoms, fully-exposed Pt-Fe clusters and FeO_x clusters are highlighted by red, green circles, dashed blue and green rectangles, respectively. Scale bar: a, 10 nm; b, 1 nm.

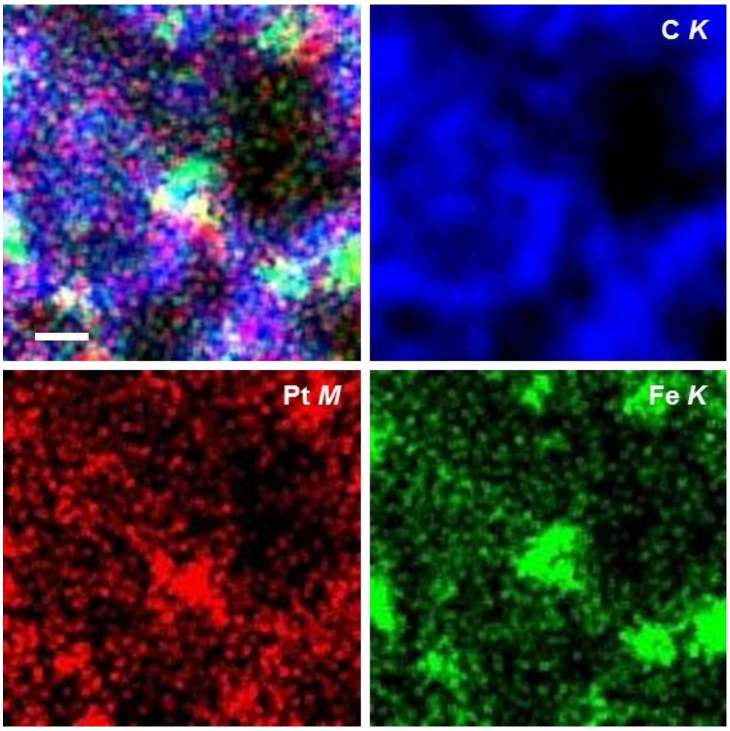

Supplementary Figure 22. EDX characterization of 0.75Pt0.3Fe/ND@G. Energy-dispersive X-ray (EDX) mapping spectroscopy of 0.75Pt0.3Fe/ND@G catalyst, scale bar = 2 nm.

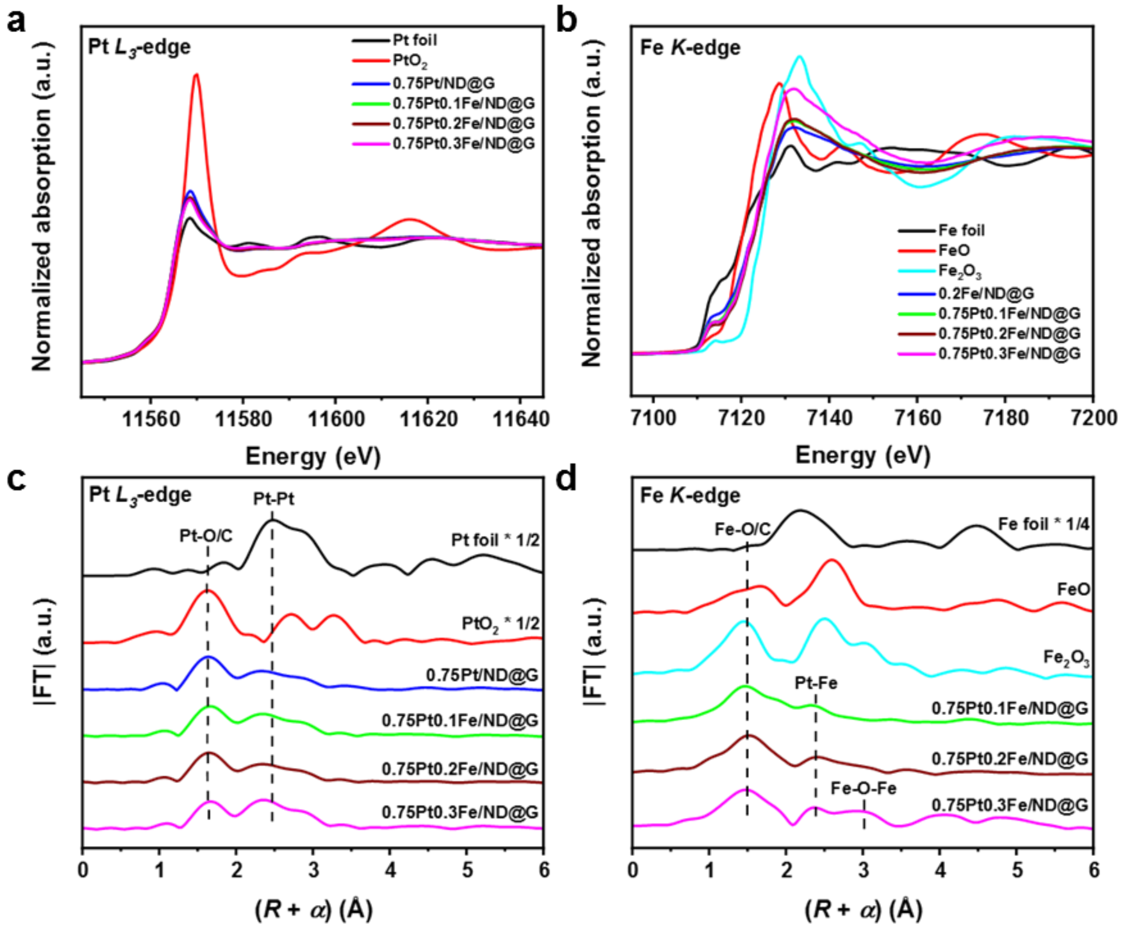

Supplementary Figure 23. X-ray absorption characterization of various PtFe/ND@G with different Fe loading.. The normalized X-ray absorption near-edge structure (XANES) spectra **(a)** and Fourier transform extended X-ray absorption fine structure (FT-EXAFS) **(c)** at the Pt L_3-edge of various PtFe/ND@G with different Fe loading; and the normalized XANES spectra **(b)** and FT-EXAFS **(d)** at the Fe K-edge of various PtFe/ND@G with different Fe loading. Reference spectra of Pt foil, PtO_2 at the Pt L_3-edge, and Fe foil, FeO, and Fe_2O_3, at the Fe K-edge are also shown for comparison.

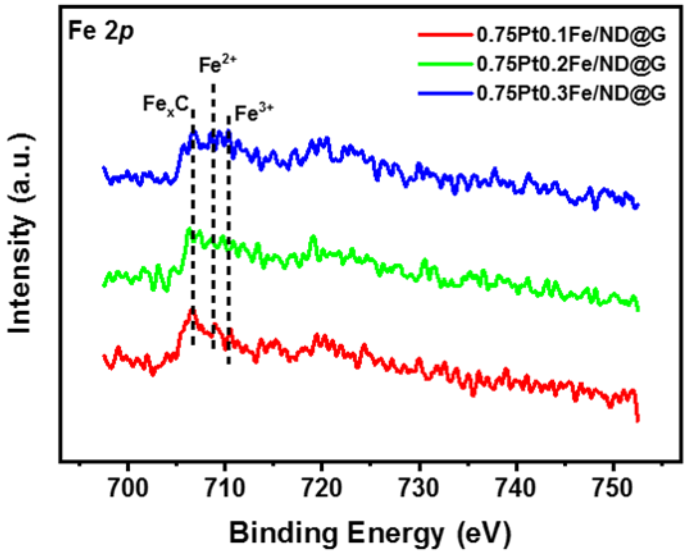

Supplementary Figure 24. XPS characterization of various PtFe/ND@G with different Fe loading. XPS spectra of various PtFe/ND@G with different Fe loading.

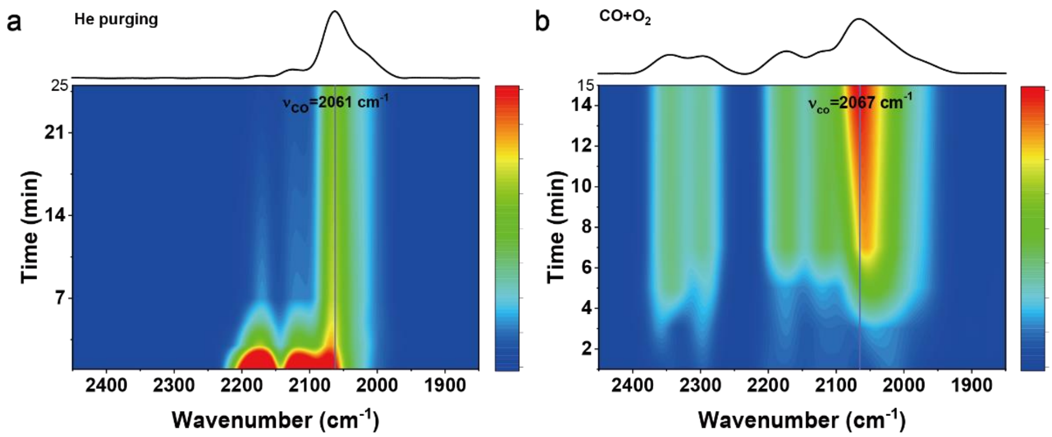

Supplementary Figure 25. CO-DRIFTS spectra of 0.75Pt/ND@G. In-situ DRIFTS study of CO adsorption during He purging **(a)** and co-adsorption CO and O_2 **(b)** on 0.75Pt/ND@G catalyst.

Supplementary Table 1. Comparison of the structure information and catalytic performance over PtFe catalysts with different Fe loading.

Catalysts	Pt Metal loading[a] (%)	Fe Metal loading[a] (%)	D[b] (%)	Temp. (^oC)	Mass specific rate ($mol_{CO}\ g_{Pt}^{-1}\ h^{-1}$)
0.75Pt/ND@G	0.83	0	92.2	30	2.87
0.75Pt0.1Fe/ND@G	0.85	0.10	92.3		4.11
0.75Pt0.2Fe/ND@G	0.82	0.18	92.1		6.19
0.75Pt0.3Fe/ND@G	0.81	0.23	91.3		3.69

a: The metal loading on various samples analysis by ICP;

b: The dispersions of Pt in various catalysts determined by CO chemisorption.

Supplementary Table 2. Fit results for the Pt edge of EXAFS spectra of various PtFe/ND@G with different Fe loading.

Sample	Paths	R (Å)	CNs	σ^2 (Å^2)	E_0 shift (eV)	R-factor
**Pt foil**	Pt-Pt	2.692	12			
	Pt-Pt	3.807	6			
**PtO_2**	Pt-O	1.999	6			
	Pt-Pt	3.136	2			
	Pt-O	3.184	2			
**0.75Pt/ND@G**	Pt-O/C	2.00(1)	1.7	0.004(1)	9.9	0.018
	Pt-Pt	2.71(1)	3.4	0.008(1)		
**0.75Pt0.1Fe/ND@G**	Pt-O/C	2.01(1)	1.4	0.003(1)	9.0	0.007
	Pt-Fe	2.59(1)	0.6	0.013(4)	7.1	
	Pt-Pt	2.71(1)	3.4	0.008(1)	7.1	
**0.75Pt0.2Fe/ND@G**	Pt-O/C	2.00(1)	1.3	0.003(1)	8.5	0.005
	Pt-Fe	2.59(4)	0.9	0.015(5)	5.0	
	Pt-Pt	2.70(1)	3.4	0.009(1)	5.0	
**0.75Pt0.3Fe/ND@G**	Pt-O/C	2.01(1)	1.0	0.002(2)	8.9	0.007
	Pt-Fe	2.60(1)	0.7	0.011(3)	6.4	
	Pt-Pt	2.72(1)	3.2	0.006(1)	6.4	
**0.75Pt0.2Fe/ND@G -used**	Pt-O/C	2.00(1)	1.7	0.003(2)	9.5	0.016
	Pt-Fe	2.60(2)	0.7	0.012(6)	7.3	
	Pt-Pt	2.72(2)	3.0	0.007(1)	7.3	

CNs, coordination numbers; R, distance between absorber and backscatter atoms; σ^2, Debye–Waller factor; $\triangle E_0$, inner potential correction. In the fits of the Pt-edge EXAFS spectra of various PtFe samples, S_0^2=0.85 fixed by Pt foil; For comparison, the interatomic distances and coordination numbers for the references (Pt foil and PtO_2) calculated from their crystallographic structures are also listed.

Supplementary Table 3. Fit results for the Fe edge of EXAFS spectra of various PtFe/ND@G with different Fe loading.

Sample	Paths	R (Å)	CNs	σ^2 ($Å^2$)	E_0 shift (eV)	R-factor
**Fe foil**	Fe-Fe	2.460	8			
	Fe-Fe	2.840	6			
**FeO**	Fe-O	2.157	6			
	Fe-Fe	3.035	6			
	Fe-Fe	3.066	6			
**Fe_2O_3**	Fe-O	1.944	3			
	Fe-O	2.114	3			
	Fe-Fe	2.893	1			
**0.2Fe/ND@G**	Fe-O/C	1.99(2)	2.7	0.008(4)	3.3	0.018
	Fe-Fe	2.50(1)	1.8	0.003(2)		
**0.75Pt0.1Fe/ND@G**	Fe-O/C	1.99(2)	3.3	0.010(4)	2.2	0.014
	Fe-Pt	2.59(2)	2.6	0.005(3)	5.0	
	Fe-Fe	3.09(2)	1.4	0.003(2)	2.2	
**0.75Pt0.2Fe/ND@G**	Fe-O/C	1.99(1)	3.9	0.011(1)	1.8	0.022
	Fe-Pt	2.60(1)	2.7	0.004(2)	2.0	
	Fe-Fe	3.09(2)	1.4	0.006(2)	1.8	
**0.75Pt0.3Fe/ND@G**	Fe-O/C	2.01(2)	4.4	0.014(2)	3.6	0.019
	Fe-Pt	2.55(1)	2.3	0.006(1)	8.0	
	Fe-Fe	3.06(1)	2.4	0.006(1)	3.6	
**0.75Pt0.2Fe/ND@G-used**	Fe-O/C	1.96(1)	4.2	0.010(1)	2.8	0.018
	Fe-Pt	2.60(4)	2.3	0.014(5)	1.5	
	Fe-Fe	3.06(2)	1.4	0.010(4)	2.8	

CNs, coordination numbers; R, distance between absorber and backscatter atoms; σ^2, Debye–Waller factor; $\triangle E_0$, inner potential correction. In the fits of the Fe-edge EXAFS spectra of various PtFe samples, S_0^2=0.8 fixed by Fe foil. For comparison, the interatomic distances and coordination numbers for the references (Fe foil, FeO, and Fe_2O_3) calculated from their crystallographic structures are also listed.

All the fitting results of Fe K-edge were performed using the ARTEMIS module built in IFEFFIT package and were done for k^2-weighted $\chi(k)$ functions with Hanning windows (dk=1 $Å^{-1}$). For all the PtFe/ND@G samples, the FT-EXAFS curves show a distinct Fe-O/C peak at the nearest neighbor shell, within the R range of 1-2 Å. And a new peak appeared at 2.55 Å assigned to the Fe-Pt path contribution, due to their different k value in the wavelet transform analysis. A low shoulder peak in the higher shell of over 3 Å was assigned to Fe-Fe coordination. Therefore, a structure model including Fe-O/C, Fe-Pt and Fe-Fe paths was used to fit the EXAFS data of these four samples. During the fittings, each of the Debye-Waller factors, coordination numbers, interatomic distances and energy shift were treated as adjustable parameters for Fe-O and Fe-Pt paths. For the Fe-Fe path, σ^2 and $\triangle E_0$ were defined as those of the Fe-O path to reduce the number of adjustable parameters.

Supplementary Table 4. Comparison of the catalytic performances over PtFe catalysts with other metal (Pt, Au, Ir) based catalysts in the PROX reaction reported in literatures.

**Catalysts**	**Noble Metal loading (%)**	**Temp. (°C)**	**Mass specific rate (mol_{CO} g_M^{-1} h^{-1})**	**Notes**
**0.75Pt0.2Fe/ND@G**	**0.75**	**30**	**6.19**	This work
0.75Pt/ND@G	0.75	30	2.14	
0.75Pt0.2Fe/SiO_2	0.75	30	0.27	
1cFe-Pt/SiO_2	3.6	27	5.21	1
2cFe-Pt/SiO_2	3.6	27	5.61	1
Pt/SiO_2	3.6	27	0.06	1
Pt/Fe-C2	2.3	27	0.26	2
Pt_1/Fe_2O_3	0.17	27	0.68	3
Au/Fe_2O_3	4.4	27	0.39	3
Au/CeO_2	0.05	40	1.1	4
1Au/CeO_2- RRCe	0.98	80	2.6	4
K-Pt/Al_2O_3	2.0	80	0.24	5
Ir/$Fe(OH)_x$	2.4	27	0.48	5
Ir/$Fe(OH)_x$	2.4	80	0.66	5

Supplementary Table 5. Comparison of the catalytic performances over PtFe catalysts with other Pt-based catalysts in the CO oxidation reaction reported in literatures.

Catalyst	Pt Metal loading (wt%)	reaction	Temp. (°C)	Specific rate×10^2 (mol_{CO} g_{Pt}^{-1} h^{-1})	TOF×10^2 (s^{-1})	Notes
**0.75Pt0.2Fe/ND@G**	**0.75**	CO oxidation	**30**	**257.1**	**15.1**	**This work**
Pt_1/FeO_x	0.17		27	43.5	13.6	[3]
Pt-SA/A-Fe_2O_3	1.2		70	125.0	6.87	[6]
Pt/Al_2O_3	2		80	-	<0.2	[7]
Pt/SiO_2	5		150	-	0.072	[8]
Pt/CeO_x/Al_2O_3	0.4		50	-	0.29	[8]
Pt/MnO_x/SiO_2	5		50	-	0.19	[8]
Pt/TiO_2	0.5		27	<0.86	<0.92	[9]
PtO_x/KLTL	1		150	-	1.2	[10]

2. Supplementary Computational Results

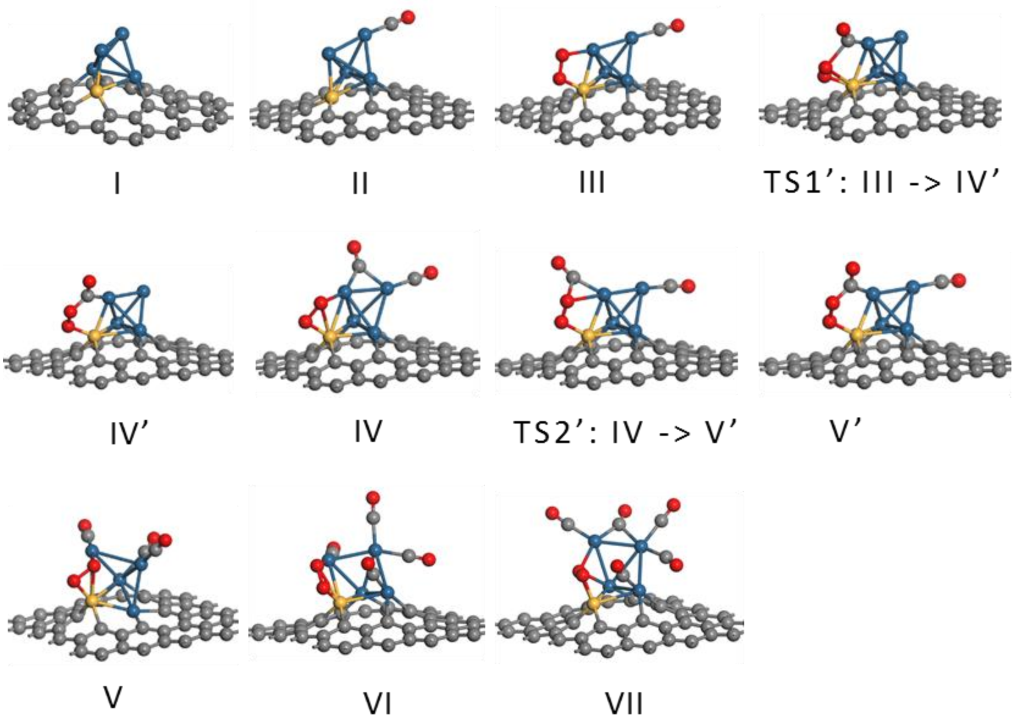

Supplementary Figure 26. Optimized geometries of Pt_4Fe_1@Gr during CO oxidation. The DFT optimized structures involved CO and O_2 adsorption on Pt_4Fe_1@Gr corresponding to Table S9.

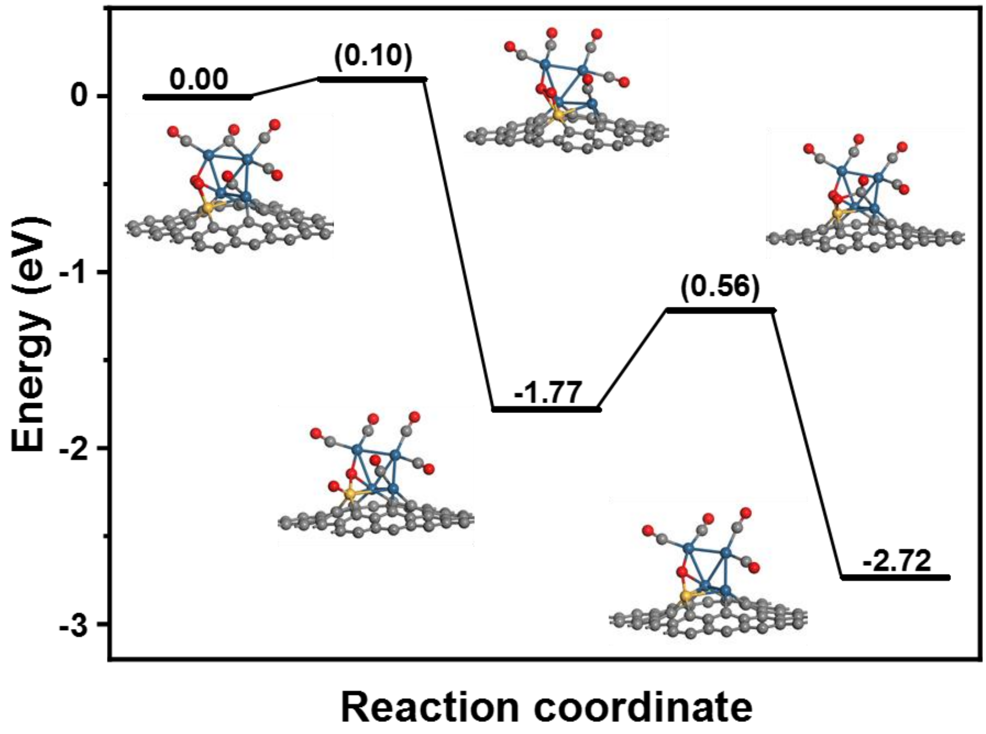

Supplementary Figure 27. Calculated potential energy diagram during the formation of Pt_4Fe_1O@Gr. DFT calculated pathways involved in the reaction of CO and O_2 to form the Pt_4Fe_1O@Gr active structure at high CO coverage.

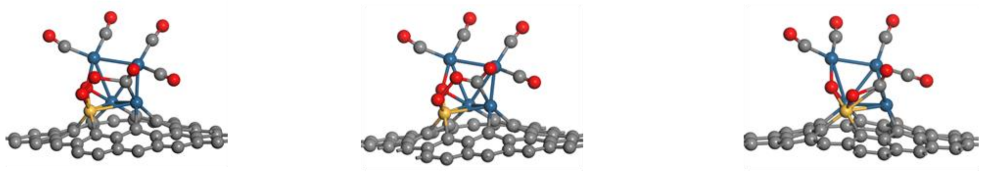

TS1: $CO^* + O_2^* \rightarrow OCOO^*$ TS2: $OCOO^* \rightarrow O^* + CO_2$ (g) TS3: $O^* + CO^* \rightarrow CO_2$ (g)

Supplementary Figure 28. Optimized geometries of transition states during CO oxidation. The structures of transition states corresponding to Table S11.

Supplementary Table 6. The structural parameters of possible Pt_4Fe_1@Gr structures.

entry	Optimized structures	CN.[a]		Energy[b](eV)
		Fe-O/C	Fe-Pt	
1	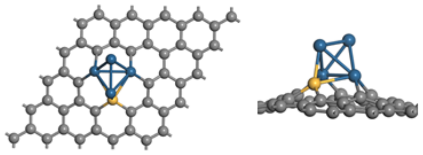	2	3	0.00
2	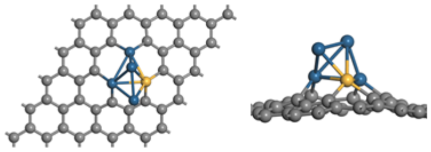	2	4	0.23
3	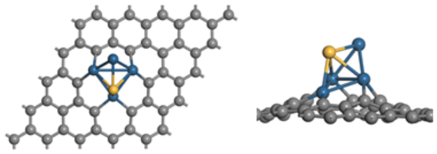	0	4	0.23
4	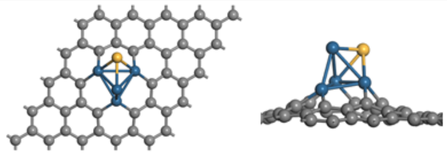	0	3	0.52

[a]CN is the coordination number; [b]the relative energy (DFT calculation) based on structure 1 are given.

Based on the experimental results including STEM and XAFS, the Pt-Fe species with $CN._{Pt\text{-}Pt}$ = 2.5 and $CN._{Fe\text{-}Pt}$ = 3.2 were atomically dispersed on the defective graphene surface. Therefore, the PtFe model, an atomic cluster with four Pt atoms and single Fe atom anchored on the graphene with four carbon vacancies (Pt_4Fe_1@Gr), was represented as the Pt-Fe species of 0.75Pt0.2Fe/NDG to discuss the CO oxidation process.

There are four possible configurations for Pt_4Fe_1@Gr structures, as shown in Table S1. Among these models, the Fe-Pt CNs in 2# and 3# models were 4, which were inconsistent with the experimental results. Besides, the Fe-C/O CN (CN=0) of the 4# model was obviously lower than the real observation value. Therefore, the 1# model is used for the subsequent DFT calculation.

Supplementary Table 7. The structures of possible optimized Pt_4Fe_1@Gr with oxygen species.

	Top view	Side view
$Pt_4Fe_1(OH)$@Gr	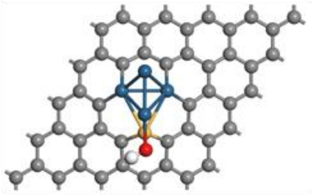	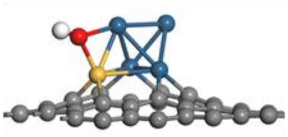
$Pt_4Fe_1(O)$@Gr	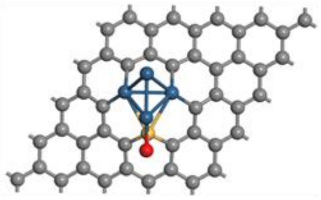	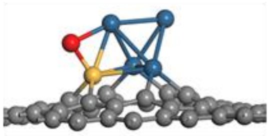

According to the experimental results about PtFe structure (the existence of Fe-C/O) and the oxidability of iron species from reported literature ($FeO(OH)_x$), we propose the Pt_4Fe_1@Gr, $Pt_4Fe_1(OH)$@Gr and $Pt_4Fe_1(O)$@Gr. It is noted that in the experiment, the catalyst was pretreated in 10% H_2/He at 400 ^oC (pressure: 1 bar) which could induce the reduction of oxygen species. Therefore, we investigated the thermodynamic stability of oxygen species on Pt_4Fe_1@Gr surface in the reduction condition (T=673.15K, P(H_2)=0.1bar).

Supplementary Table 8. The energy change of reduction reactions.

Reaction	ΔE (eV)	ΔG (eV)
$Pt_4Fe_1(O)@Gr + H_2(gas) \text{->} Pt_4Fe_1@Gr + H_2O(gas)$	-0.28	-0.75
$Pt_4Fe_1(OH)@Gr + 0.5\ H_2(gas) \text{->} Pt_4Fe_1@Gr + H_2O(gas)$	0.47	-0.56

It was observed from DFT calculation that ΔG of two reactions are below zero, indicating that $Pt_4Fe_1(OH)@Gr$ and $Pt_4Fe_1(O)@Gr$ could be readily reduced to $Pt_4Fe_1@Gr$ under the reduction condition. Therefore, the $Pt_4Fe_1@Gr$ model is used to investigate the CO oxidation process.

Supplementary Table 9. The detailed energy barriers (E_a) and reaction energies (ΔE) involved in CO and O_2 adsorption on Pt_4Fe_1@Gr.

Reactions	ΔE	E_a
I -> II: CO(g) -> CO*	-2.42	
II -> III: CO* + O_2(g) -> CO* + O_2*	-1.75	
III -> IV': CO* + O_2* -> OCOO*	1.32	1.65
III -> IV: CO* + O_2* + CO(g) -> 2CO* + O_2*	-1.18	
IV -> V': 2CO* + O_2* -> OCOO* + CO*	0.72	1.06
IV -> V: 2CO* + O_2* + CO(g) -> 3CO* + O_2*	-1.36	
V -> VI: 3CO* + O_2* + CO(g) -> 4CO* + O_2*	-1.20	
VI -> VII: 4CO* + O_2* + CO(g) -> 5CO* + O_2*	-0.86	

Supplementary Table 10. The DFT calculated pathways in forming the Pt_4Fe_1O@Gr active structure.

Reactions	ΔE	E_a
5CO* + O_2* -> 5CO* + 2O*	-1.77	0.10
5CO* + 2O* -> 4CO* + O* + CO_2(g)	-0.95	0.56

Supplementary Table 11. The energies of species in the proposed CO oxidation reaction on Pt_4Fe_1O@Gr.

species	ΔE	E_a
i: CO(g) + O_2(g)		
ii: O_2*	-1.25	
iii: CO* + O_2*	-0.36	
TS1: CO* + O_2* -> OCOO*		0.21
iv: OCOO*	-0.03	
TS2: OCOO* -> O* + CO_2 (g)		0.46
v:O*	-2.53	
vi: O* + CO*	-1.34	
TS3: O* + CO* -> CO_2 (g)		0.76
i: CO_2 (g)	-1.02	

3. Supplementary References

1. Cao, L. N. *et al.* Atomically dispersed iron hydroxide anchored on Pt for preferential oxidation of co in H2. *Nature* **565**, 631-635 (2019).
2. Qiao, B. T. *et al.* Ferric oxide-supported Pt subnano clusters for preferential oxidation of CO in H2-rich gas at room temperature. *ACS Catal.* **4**, 2113-2117 (2014).
3. Qiao, B. *et al.* Single-atom catalysis of CO oxidation using Pt1/FeOx. *Nat. Chem.* **3**, 634-641 (2011).
4. Qiao, B. T. *et al.* Highly efficient catalysis of preferential oxidation of CO in H2-rich stream by gold single-atom catalysts. *ACS Catal.* **5**, 6249-6254 (2015).
5. Lin, J. *et al.* Design of a highly active Ir/Fe(OH)x catalyst: Versatile application of Pt-group metals for the preferential oxidation of carbon monoxide. *Angew. Chem. Int. Ed.* **51**, 2920-2924 (2012).
6. Chen, W. *et al.* Strong electronic interaction of amorphous Fe2O3 nanosheets with single-atom Pt toward enhanced carbon monoxide oxidation. *Adv. Funct. Mater.* **29**, 1904278 (2019).
7. Minemura, Y. *et al.* Preferential CO oxidation promoted by the presence of H2 over K-Pt/Al2O3. *Chem. Commun.*, 1429-1431 (2005).
8. Mergler, Y. J., vanAalst A., vanDelft J., Nieuwenhuys B. E. CO oxidation over promoted Pt catalysts. *Appl. Catal. B* **10**, 245-261 (1996).
9. Bamwenda, G. R., Tsubota S., Nakamura T., Haruta M. The influence of the preparation methods on the catalytic activity of platinum and gold supported on TiO2 for CO oxidation. *Catal. Lett.* **44**, 83-87 (1997).
10. Kistler, J. D. *et al.* A single-site platinum CO oxidation catalyst in zeolite KLTL: Microscopic and spectroscopic determination of the locations of the platinum atoms. *Angew. Chem. Int. Ed.* **53**, 8904-8907 (2014).
